# Post-vertebroplasty lumbar spondylodiscitis due to *Cutibacterium modestum*

**DOI:** 10.3389/fmed.2026.1905143

**Published:** 2026-09-16

**Authors:** Liyang Wu, Yongzhen Bao, Jiahao Pu, Lijuan Zhang, Jinshan Huang, Xinhua Xi, Lincong Luo

**Affiliations:** 1 The Affiliated Yuebei People’s Hospital of Shantou University Medical College, Shaoguan, Guangdong, China; 2 Yue Bei People’s Hospital Postdoctoral Innovation Practice Base, Southern Medical University, Guangzhou, Guangdong, China; 3 School of Basic Medical Sciences, Fujian Medical University, Fuzhou, Fujian, China

**Keywords:** Cutibacterium modestum, metagenomic sequencing, pathogen attribution, percutaneous vertebroplasty, postoperative spinal infection, spondylodiscitis

## Abstract

Percutaneous vertebroplasty-associated spinal infection is uncommon but may cause progressive vertebral destruction and can mimic tuberculous spondylitis on imaging. The detection of low-virulence skin commensals such as *Cutibacterium* species in deep specimens also creates a diagnostic challenge because contamination and true infection must be distinguished. We report a 78-year-old man who developed recurrent progressive low back pain shortly after percutaneous vertebroplasty for an L2 compression fracture. Baseline computed tomography (CT) and contrast-enhanced magnetic resonance imaging (MRI) showed destructive spondylodiscitis centered at L2/3, with adjacent vertebral body and disc-space involvement, paravertebral inflammatory extension, and bilateral psoas abscess-like collections, initially suggesting tuberculous spondylitis. However, molecular testing for the *Mycobacterium tuberculosis* complex and targeted sequencing for mycobacterial drug-resistance genes were negative, and histopathology demonstrated acute and chronic inflammatory necrotic changes without typical tuberculous granulomas. Metagenomic next-generation sequencing of aspirated fluid identified a high abundance of *Cutibacterium modestum*, which was subsequently detected again in lesion tissue and peripheral blood by targeted high-throughput sequencing. Given the repeated detection in deep-site specimens, exclusion of tuberculosis and other competing diagnoses, compatible clinical and imaging findings, and improvement after targeted antimicrobial therapy and percutaneous stabilization, *C. modestum* was considered the most probable causative pathogen. These findings suggest that pathogen attribution in post-vertebroplasty spinal infection should rely on integrated clinical, microbiological, pathological, and therapeutic evidence rather than on imaging findings alone. Integrated clinico-microbiological assessment may improve pathogen attribution for rare low-virulence organisms in postoperative spinal infection.

## Introduction

Percutaneous vertebroplasty (PVP) has become an established treatment for osteoporotic vertebral compression fractures because it is minimally invasive, provides rapid pain relief, and facilitates early recovery. Although postoperative infection is uncommon, it can lead to serious complications when it occurs. More importantly, such infections may be associated with delayed diagnosis, progressive local bone destruction, repeat surgical intervention, and poor functional recovery. Accordingly, postoperative infection remains one of the most serious complications associated with PVP (1, 2). However, diagnosis is further complicated by the fact that postoperative PVP infections often lack specific clinical manifestations in the early stage. In many cases, patients present only with persistent or progressively worsening low back pain, whereas systemic signs of infection, such as fever, may be absent (3). Moreover, postoperative imaging changes, the natural evolution of the fracture, and coexisting degenerative changes may obscure the underlying disease process, thereby contributing to delayed diagnosis. Therefore, in patients with persistent pain after PVP, the central challenge is not merely to identify imaging abnormalities, but to determine whether these findings represent a true deep infection that requires targeted microbiological evaluation and treatment.

This diagnostic dilemma is particularly pronounced in infections caused by low-virulence pathogens. In recent years, the clinical relevance of the genus *Cutibacterium* in implant-associated, osteoarticular, and spinal infections has become increasingly recognized, particularly in indolent postoperative spine infections (4). However, because *Cutibacterium* species are also common components of the normal skin microbiota, distinguishing contamination from true infection remains challenging (5, 6). In 2020, *Cutibacterium modestum* was formally recognized as a distinct species. Subsequent studies further showed that it is identical to the previously described “*Propionibacterium humerusii*” and may be misidentified as *C. acnes* under routine laboratory conditions (6, 7). Although published case reports remain scarce, the available evidence suggests that this organism can cause vertebral osteomyelitis and other deep-seated infections (8). Thus, determining the clinical significance of *C. modestum* remains an important diagnostic challenge.

The diagnosis of postoperative spinal infection may be challenging, particularly when conventional culture is negative or inconclusive. In this setting, molecular pathogen testing, including metagenomic next-generation sequencing and targeted high-throughput sequencing, may facilitate the detection of low-biomass or difficult-to-culture organisms (9, 10). However, for low-virulence skin commensals, molecular detection alone does not establish causality and should be interpreted together with specimen source and the clinical context (11). At present, post-PVP spinal infection associated with *C. modestum* remains rarely reported. Accordingly, we report a case of healthcare-associated post-PVP lumbar spondylodiscitis due to *C. modestum* and discuss the challenges of pathogen attribution for this low-virulence organism (Figure 1).

**Figure 1:**
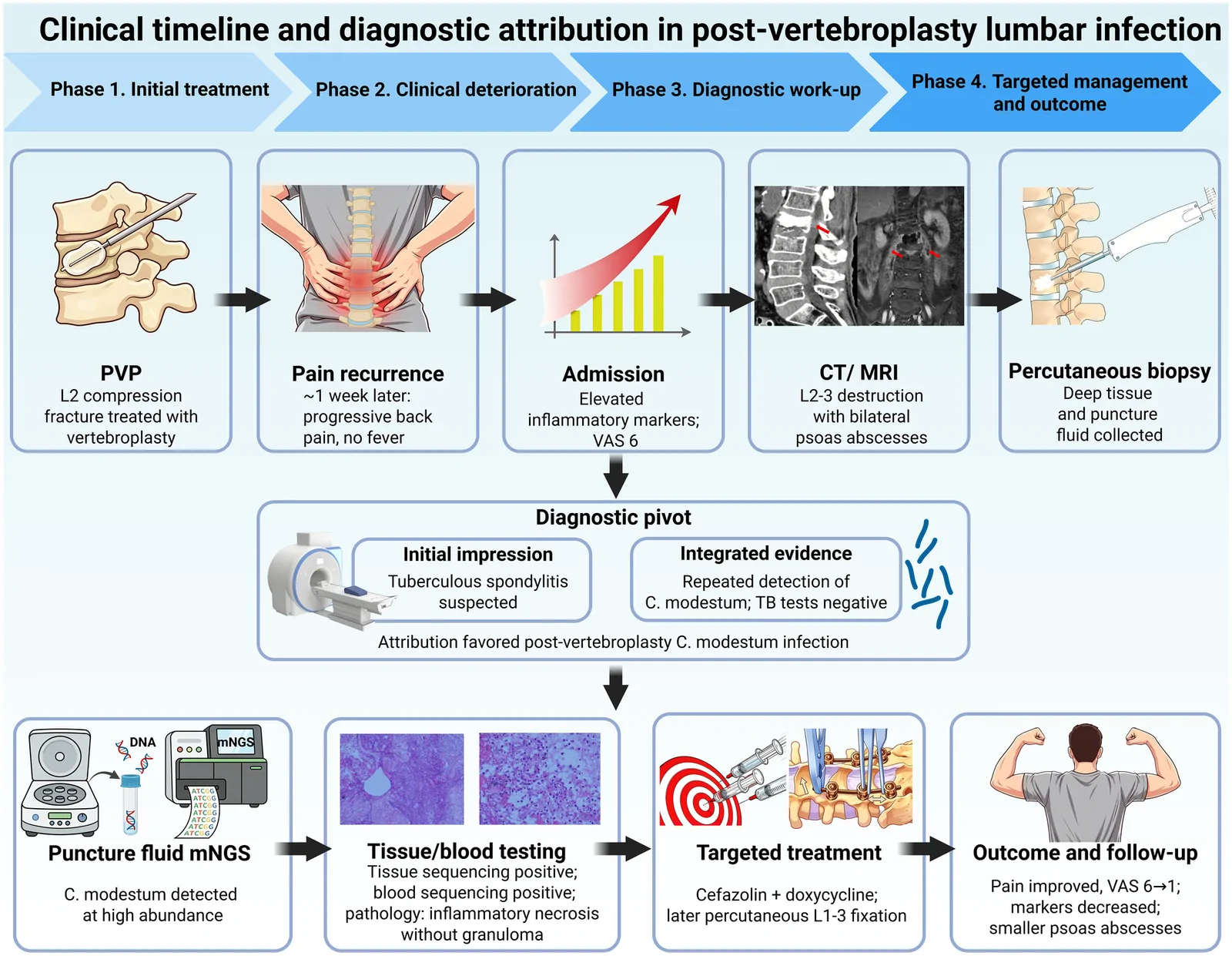
Clinical timeline and etiologic attribution in post-vertebroplasty destructive lumbar spondylodiscitis. After PVP for an L2 compression fracture, the patient developed recurrent progressive low back pain and was admitted approximately 1 month later. CT and MRI showed destructive L2/3 spondylodiscitis with bilateral psoas abscesses, initially suggesting tuberculous spondylitis. Percutaneous biopsy enabled deep lesion sampling. *C. modestum* was detected by molecular testing in two local lesion-derived samples, including aspirated fluid and lesion tissue, and was also detected in peripheral blood by targeted high-throughput sequencing. It was not isolated by conventional culture. Tuberculosis-related testing was negative and histopathology showed no typical granulomas. Integrated evidence favored *C. modestum* as the most likely pathogen. After targeted antimicrobial therapy and L1–L3 fixation, pain and inflammatory markers improved, and the psoas abscesses decreased.

## Case presentation

A 78-year-old man with a history of type 2 diabetes presented to our hospital. He had previously undergone PVP at another hospital for an L2 vertebral compression fracture. Initially, his symptoms improved briefly after the procedure, and he was discharged. However, approximately 1 week later, low back pain recurred and progressively worsened until he became bedridden and unable to walk. Notably, he remained afebrile throughout the course of illness. Approximately 1 month after PVP, he presented to our hospital because of marked worsening of low back pain. On admission, he was afebrile and normotensive, with a temperature of 36.5 °C and blood pressure of 105/78 mmHg. He had marked tachycardia, with a heart rate of 140 beats/min, whereas the respiratory rate was 20 breaths/min without clinically significant tachypnea. Physical examination showed marked lower back tenderness and restricted lumbar motion, without obvious neurologic deficits. In addition, the visual analogue scale (VAS) pain score was 6 on a 0–10 scale, where 0 indicates no pain and 10 indicates the worst imaginable pain (12). Laboratory testing further suggested a mild inflammatory response. Specifically, the white blood cell count was 9.88 × 10⁹/L, the neutrophil percentage was 78.40%, C-reactive protein was 1.43 mg/dL, erythrocyte sedimentation rate was 57 mm/h, procalcitonin was 0.381 ng/mL, and interleukin-6 was 17.01 pg/mL. Routine peripheral blood culture remained negative, and no staphylococcal species was isolated from blood culture. Lumbar CT showed post-vertebroplasty changes at L2, together with irregular low-density osteolytic/destructive changes involving the L2 and L3 vertebral bodies and the L2/3 disc space. These abnormalities were accompanied by swelling of both psoas muscles and the paravertebral soft tissues (Figures 2A–C). Lumbar MRI was performed using sagittal T1-weighted imaging, sagittal T2-weighted/fat-suppressed imaging, and gadolinium-based contrast-enhanced T1-weighted imaging in sagittal, coronal, and axial planes. Contrast-enhanced MRI demonstrated abnormal enhancement of the L2 and L3 vertebral bodies, the L2/3 disc space, and adjacent paravertebral soft tissues, along with bilateral psoas abscess-like collections (Figures 2D–G).

**Figure 2:**
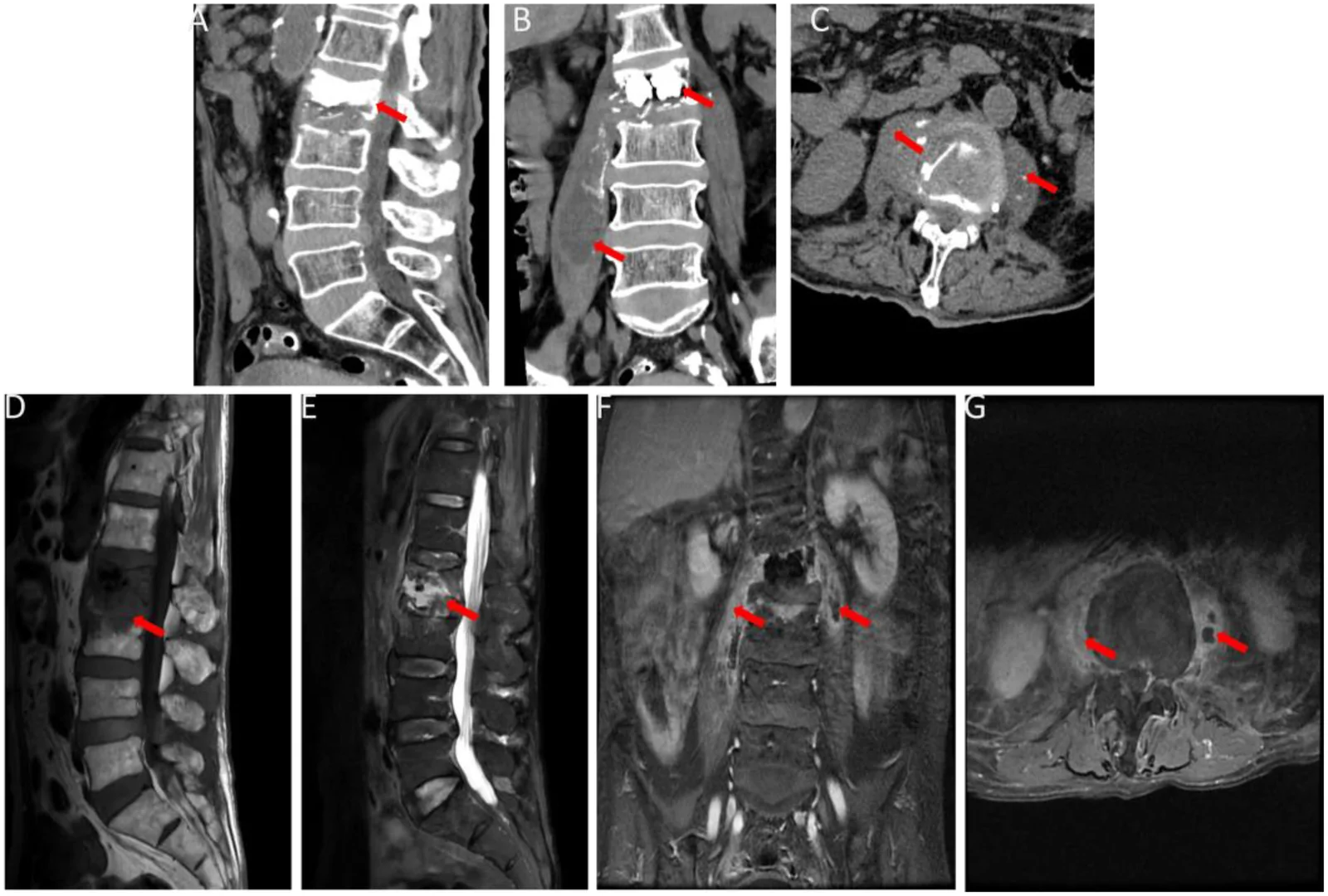
Baseline imaging findings of post-vertebroplasty destructive lumbar spondylodiscitis. **(A–C)** Baseline CT images showing post-vertebroplasty changes at L2, with retained bone cement and irregular destruction involving the adjacent L2/3 endplates and disc space. Paravertebral soft-tissue swelling and bilateral psoas involvement are also observed. **(D–G)** Baseline MRI images showing abnormal signal intensity and enhancement around the L2/3 level, involving the adjacent vertebral bodies, intervertebral disc space, and paravertebral soft tissues. Bilateral psoas abscess-like collections are evident on coronal and axial contrast-enhanced images. Overall, the combined CT and MRI findings indicate destructive lumbar spondylodiscitis centered at L2/3, initially mimicking tuberculous spondylitis. Red arrows indicate the principal sites of vertebral destruction, disc-space involvement, paravertebral extension, and psoas abscess-like collections.

Therefore, because the imaging strongly suggested infection and pathogen identification was required, a bilateral L2 transpedicular percutaneous biopsy was performed under x-ray guidance after admission to differentiate the lesion from tuberculous spondylitis. Tissue samples were obtained for histopathologic examination, *Mycobacterium tuberculosis* complex nucleic acid testing, and targeted sequencing for mycobacterial drug-resistance genes. At the same time, aspirated fluid was collected for aerobic and anaerobic culture, as well as metagenomic testing for pathogen detection. Meanwhile, empiric antimicrobial therapy with moxifloxacin plus rifampin was initiated. The day after biopsy, metagenomic analysis of the aspirate identified a high abundance of *Cutibacterium modestum*, with 6,642 sequence reads and a relative abundance of 99.903%. In contrast, targeted sequencing for the *Mycobacterium tuberculosis* complex and drug-resistance genes was negative.

Subsequently, conventional culture of the aspirate grew *Staphylococcus capitis subsp. capitis. S. capitis* is a coagulase-negative staphylococcus and has been reported in invasive or device-associated infections, including device-related bone and joint infections and osteomyelitis (13). Targeted high-throughput sequencing of the lesion tissue again detected *C. modestum*, with 4,917 sequence reads and a relative abundance of 93.9%. A routine peripheral blood culture was performed once and yielded no growth of *C. modestum* or *staphylococcal* species. In contrast, targeted high-throughput sequencing of peripheral blood detected *C. modestum.* Histopathologic examination showed that the submitted tissue was predominantly necrotic and infiltrated by neutrophils, lymphocytes, and plasma cells, consistent with acute and chronic inflammatory necrotic change. In contrast, no typical tuberculous granulomatous features were identified (Figure 3).

**Figure 3:**
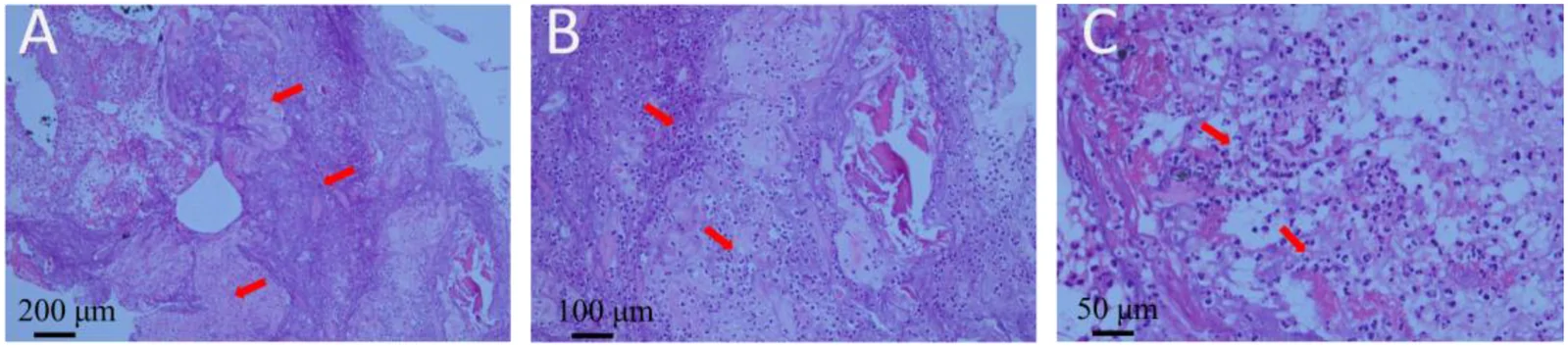
Histopathological examination of the vertebral lesion. **(A)** Low-power view showing predominantly necrotic tissue with diffuse inflammatory involvement. **(B)** Intermediate-power view demonstrating inflammatory necrotic changes with mixed inflammatory cell infiltration. **(C)** High-power view showing prominent infiltration of neutrophils, lymphocytes, and plasma cells, consistent with acute and chronic inflammatory necrotic change. Red arrows indicate representative areas of necrosis and inflammatory cell infiltration. No typical tuberculous granulomatous structures are identified. Hematoxylin and eosin staining. Scale bars: A, 200 μm; B, 100 μm; C, 50 μm.

*C. modestum* was detected by molecular testing in two local lesion-derived samples, including aspirated fluid and lesion tissue, and was also detected in peripheral blood by targeted high-throughput sequencing. Following consultation with the Pharmacy Department, the antimicrobial regimen was revised. Specifically, the revised regimen consisted of intravenous cefazolin sodium 2 g every 6 h combined with oral doxycycline 0.1 g twice daily. At the same time, insulin therapy was initiated to optimize glycemic control. Subsequently, the patient showed progressive clinical improvement, accompanied by reductions in pain and inflammatory markers. Nevertheless, because functional limitation persisted and local structural stability remained a concern, percutaneous L1–L3 pedicle screw fixation was performed after initial infection control had been achieved. The same antimicrobial regimen was continued postoperatively (Figures 4A–D).

**Figure 4:**
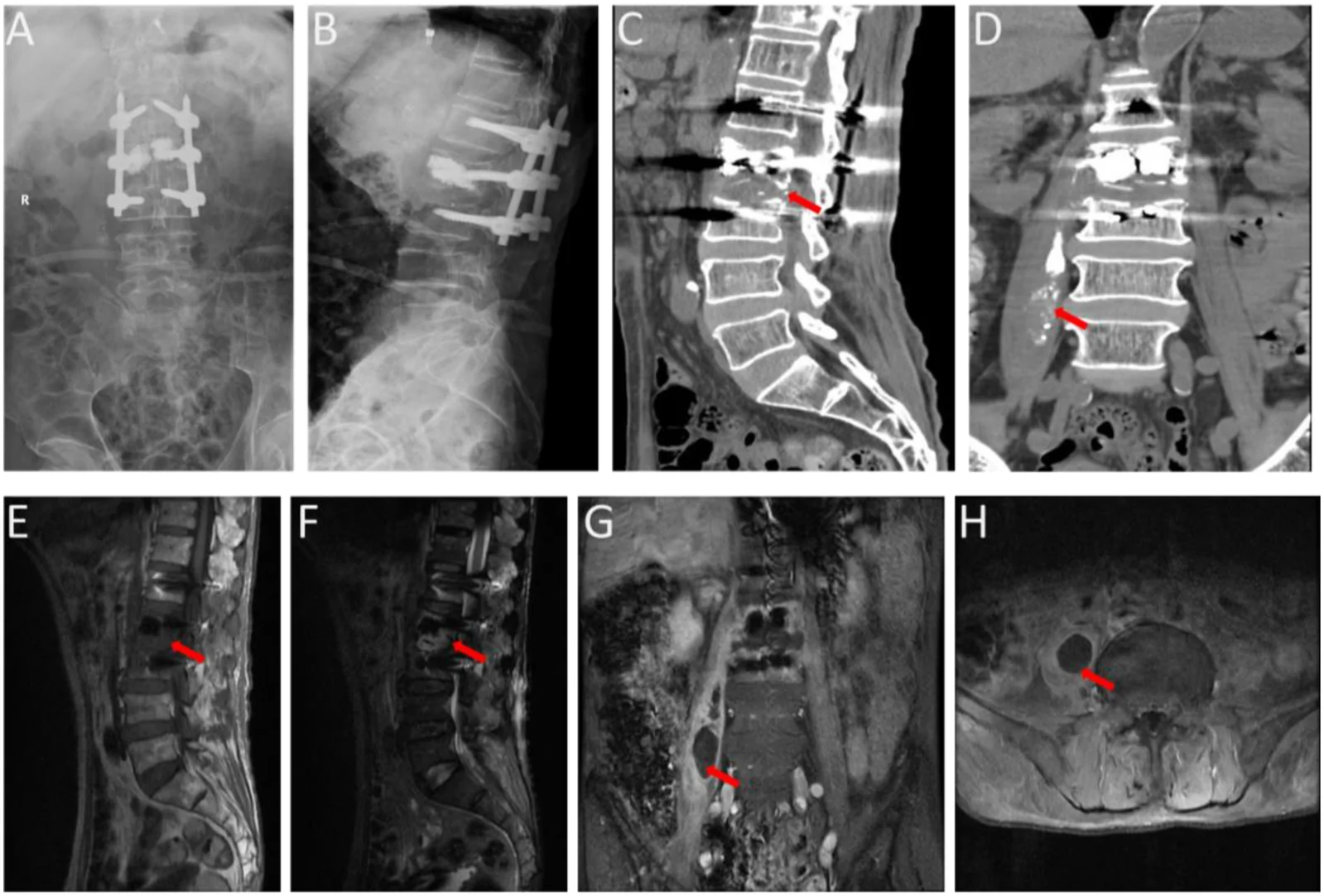
Follow-up imaging after antimicrobial therapy and percutaneous L1–L3 fixation. **(A,B)** Anteroposterior and lateral radiographs showing percutaneous L1–L3 pedicle screw fixation after initial infection control, with retained bone cement at the previously treated L2 vertebral body. **(C,D)** Sagittal and coronal CT images demonstrating persistent destructive changes around the L2/3 level, involving the adjacent vertebral endplates and disc space, with residual paravertebral and psoas involvement. **(E,F)** Sagittal MRI images showing persistent abnormal signal changes at the infected vertebral level and adjacent disc space after treatment. **(G,H)** Coronal and axial contrast-enhanced MRI images showing residual paravertebral inflammatory extension and psoas abscess-like changes, with reduction of the abscess component compared with the initial imaging. These findings illustrate that bone destruction may persist or appear more prominent on short-term follow-up despite clinical improvement and reduction of soft-tissue abscesses. Red arrows indicate the principal residual lesions.

Before discharge, low back pain had improved substantially, with the VAS score decreasing from 6 to 1. However, follow-up contrast-enhanced MRI showed increased bone destruction in the L2 and L3 vertebral bodies compared with prior imaging. At the same time, the bilateral psoas abscesses had decreased in size (Figures 4G,H). Meanwhile, both clinical symptoms and inflammatory markers continued to improve.

## Discussion

The significance of this case lies not merely in reporting a rare *Cutibacterium* species, but in highlighting the challenge of pathogen attribution in healthcare-associated post-PVP lumbar spondylodiscitis caused by a low-virulence skin commensal. *Tuberculosis* was considered only as an initial differential diagnosis because of destructive spondylodiscitis with paravertebral and bilateral psoas involvement. However, tuberculosis-related molecular tests were negative, and histopathology showed no typical granulomatous inflammation. *Brucella* spondylodiscitis should also be considered in the differential diagnosis of subacute or chronic spondylodiscitis, particularly in endemic regions; however, in the present case, the close temporal relationship with PVP, the healthcare-associated context, and repeated molecular detection of *C. modestum* in lesion-derived specimens favored post-procedural infection as the most plausible diagnosis (14). The diagnosis in this case should not rest on a single positive specimen or an isolated positive test result. Instead, it should be established through a stepwise framework for pathogen attribution. First, the patient had undergone PVP. After this procedure, he developed progressive pain, reduced mobility, and radiographic evidence of destructive spinal involvement, together with paraspinal infection extending into both psoas muscles. Taken together, this clinical picture was highly consistent with deep spinal infection. Second, although spinal tuberculosis was the leading competing diagnosis on imaging, it was not supported by either microbiological or pathological evidence. Nucleic acid testing and targeted sequencing for the *Mycobacterium tuberculosis* complex were negative, whereas histopathology showed necrotic tissue infiltrated by neutrophils, lymphocytes, and plasma cells, without the granulomatous features expected in tuberculosis. In addition, routine culture yielded *Staphylococcus capitis subsp. capitis* on a single occasion. Previous studies have shown that *S. capitis* can cause clinically relevant infections, particularly in device-associated or osteoarticular settings (13). Nevertheless, in the present case, the evidentiary value of this single culture-positive result was weaker than that of repeated *C. modestum* detection in lesion aspirate, lesion tissue, and peripheral blood. Notably, *C. modestum* was identified in both key lesion-derived specimens—aspirate and tissue—and was also detected in peripheral blood, providing much stronger support for pathogen attribution than a single culture-positive coagulase-negative staphylococcus. Importantly, the Infectious Diseases Society of America (IDSA) guidelines for native vertebral osteomyelitis in adults state that *Cutibacterium/Propionibacterium* species and coagulase-negative staphylococci identified in image-guided aspiration samples should be interpreted cautiously because they may represent either skin contaminants or true pathogens (5). Accordingly, pathogen attribution should rely on integrated microbiological, pathological, and clinical evidence rather than on a single positive result alone. Similarly, studies of *C. acnes* vertebral osteomyelitis suggest that when *Cutibacterium spp.* are detected only once and are unsupported by compatible clinical or imaging findings, contamination is likely; by contrast, prior spinal procedures, compatible imaging, and supportive pathology substantially increase the likelihood of true pathogenicity (15). Therefore, the clinical significance of *C. modestum* in this case did not arise from mere detection, but from an integrated body of evidence that included repeated detection in deep-site specimens, concordance with the patient's procedural history and clinical context, exclusion of competing diagnoses, and a supportive treatment response.

The initial suspicion of tuberculosis was not driven simply by “similar” imaging findings. Rather, it reflected a more fundamental limitation: in spinal infection, imaging patterns primarily mirror the extent of tissue destruction and inflammatory spread, rather than pathogen-specific characteristics, and recent reviews have emphasized the substantial overlap between infectious spondylodiscitis, tuberculous spondylitis, and its mimickers (16). Classically, tuberculous spondylitis is characterized radiographically by endplate destruction, disc space involvement, paravertebral soft-tissue enhancement, and psoas abscess formation. However, low-virulence, slowly progressive *Cutibacterium* infections may present in a similar fashion, with localized pain, a relatively mild systemic inflammatory response, and progressive local tissue destruction. Indeed, previous studies and recent reviews have examined spinal infections caused by *Cutibacterium* species, particularly in indolent postoperative or implant-associated spine infections (4). Collectively, these studies indicate that such infections often follow an indolent course, presenting with pain, radiographic destruction, and implant- or procedure-related complications rather than overt systemic manifestations such as high fever or sepsis (17). Compared with the recent literature, the present case is consistent with the indolent presentation of Cutibacterium-related spinal infection, but is distinctive because it developed after PVP, showed tuberculosis-like imaging findings, and involved repeated detection of *C. modestum* in lesion aspirate, lesion tissue, and peripheral blood. Furthermore, because the infection was centered on the vertebrae and disc space adjacent to the operative site, procedure-related inoculation represents a reasonable clinical inference. Nevertheless, in the absence of strain typing or other higher-level confirmatory evidence, this inference should be interpreted with caution. Accordingly, the central message of this case is not simply that tuberculosis may be over-suspected, but that tuberculosis-like imaging findings do not amount to microbiological confirmation of tuberculosis. Therefore, in postoperative settings involving implanted material and host susceptibility, imaging-based impressions must ultimately be substantiated by microbiological evidence.

In this context, the value of molecular pathogen testing lies not merely in improving detection rates, but in clarifying whether a detected organism is likely to be clinically relevant (18). However, because molecular techniques may also detect contaminants or colonizing organisms, their results should be interpreted together with specimen source, clinical context, imaging findings, histopathology, and treatment response. For low-virulence organisms such as *Cutibacterium*, which combine commensal behavior with pathogenic potential, a single positive result is insufficient to support clinical attribution. More importantly, the key considerations are whether the result is derived from deep lesion specimens, whether it is reproducible across samples, whether it can be interpreted in relation to other microbiological findings, and whether it is concordant with the clinical course. In the 2023 case report of *C. modestum* spondylitis, the persuasiveness of metagenomic analysis derived from more than simple organism detection. Specifically, the use of decontamination strategies, including duplicate samples, negative controls, and differential abundance analysis, was critical in showing that detection of *C. modestum* in lesion tissue did not represent a random background signal (11). Similarly, in the present case, *C. modestum* was not identified in a single specimen alone, but was consistently detected in 2 deep lesion-derived samples—aspirate and tissue—and was further supported by molecular detection in peripheral blood. Accordingly, from a rigorous evidentiary perspective, *C. modestum* is best regarded as the most probable causative pathogen in this case. Nevertheless, because no viable *C. modestum* isolate was obtained, alternative diagnoses should be considered. First, spinal tuberculosis was an important competing diagnosis because the patient showed tuberculosis-like imaging findings, including vertebral destruction, disc-space involvement, paravertebral extension, and bilateral psoas abscesses. However, tuberculosis-related molecular tests were negative, and histopathology showed no typical granulomatous inflammation. Second, infection caused by the coagulase-negative staphylococcus detected in the aspirate culture was also considered. However, this organism was recovered from only one culture, whereas *C. modestum* was repeatedly detected in lesion aspirate, lesion tissue, and peripheral blood. Third, a polymicrobial postoperative infection cannot be completely excluded, particularly in the setting of prior vertebroplasty and bone cement implantation. However, the repeated high-abundance molecular detection of *C. modestum*, together with clinical improvement after targeted antimicrobial therapy, made *C. modestum* the most plausible primary pathogen. Finally, noninfectious post-vertebroplasty inflammatory or mechanical changes were unlikely to fully explain the bilateral psoas abscess-like collections, progressive vertebral destruction, inflammatory marker elevation, and microbiological findings. A key practical lesson from this case is that treatment response should not be assessed solely on the basis of short-term changes in osseous imaging. Following treatment, the patient experienced substantial pain relief, together with improvement in inflammatory markers. In addition, follow-up imaging showed a reduction in the size of the bilateral psoas abscesses. However, MRI still demonstrated marked vertebral destruction and marrow edema compared with earlier examinations. Accordingly, defining treatment failure solely on the basis of apparently worsening bone imaging may lead to misinterpretation of the disease course. Importantly, the IDSA guidelines state that in patients with vertebral osteomyelitis whose symptoms and inflammatory markers are improving, short-term follow-up MRI should not be used routinely to assess treatment response; even when bone imaging appears worse at 4–6 weeks, treatment should not be considered a failure in the absence of parallel clinical or laboratory deterioration (5). Moreover, subsequent studies have reported a similar lack of correlation between follow-up MRI findings and clinical recovery in spinal infection. Specifically, soft-tissue inflammation and abscesses often improve early, whereas bone and disc destruction may persist for months and may even appear to progress despite adequate infection control (19–21). Therefore, in the present case, reduction of the psoas abscesses, relief of pain, and decline in inflammatory markers were more reliable indicators of treatment response than short-term progression on bone imaging. This point is particularly relevant in postoperative spinal infection, where overinterpretation of bony imaging abnormalities may prompt unnecessary escalation of antimicrobial therapy or additional surgical intervention.

Taken together, although multi-site molecular detection, exclusion of competing diagnoses, and the observed treatment response strengthen the attribution of *Cutibacterium modestum* as the causative pathogen in this case, several limitations still warrant caution. First, no *C. modestum* isolate was obtained, which precluded higher-level orthogonal confirmation, such as culture-based 16S ribosomal RNA (rRNA) sequencing. Second, follow-up remains relatively short and is insufficient to evaluate long-term infection control. Therefore, this case should not be interpreted as definitive proof of the pathogen's virulence or as absolute exclusion of all alternative etiologies. Rather, it is better understood as a high-confidence clinical microbiology framework supporting *C. modestum* as the most likely causative pathogen in post-PVP destructive lumbar spinal infection with tuberculosis-like imaging findings. More importantly, this case underscores that the diagnosis of postoperative spinal infection should not rely exclusively on radiologic impressions. For low-virulence skin commensals such as *Cutibacterium*, repeated detection in deep lesion specimens, together with supportive host context, exclusion of competing diagnoses, and treatment response, should caution clinicians against dismissing these organisms *a priori* as mere contaminants. In this respect, the value of this case lies not only in expanding the clinical spectrum of postoperative spinal infection associated with *C. modestum*, but also in providing a useful clinical microbiology framework for assessing the pathogenicity of rare, low-virulence organisms and for guiding diagnosis and management in similar cases.

## Conclusion

This case highlights the diagnostic challenge posed by low-virulence skin commensals in healthcare-associated post-PVP spinal infection. For organisms such as Cutibacterium, pathogen attribution should be based on an integrated assessment of repeated detection in clinically relevant specimens, the clinical and pathological context, and treatment response, rather than dismissing the organism as contamination. In this setting, metagenomic next-generation sequencing and targeted high-throughput sequencing can provide valuable complementary evidence for identifying low-biomass or difficult-to-culture pathogens in postoperative osteoarticular infection and spondylodiscitis, particularly when conventional culture is negative or inconclusive. However, molecular results should be interpreted in conjunction with deep-site sampling, histopathology, clinical context, and treatment response. Taken together, this integrated approach provides a practical basis for accurate diagnosis and appropriate management in similar cases.

## Data Availability

The clinical information and diagnostic findings supporting this case report are included in the article. Access to underlying patient-level data is subject to the confidentiality provisions of the consent documentation. Data inquiries should be directed to the corresponding authors, Xinhua Xi (56249042@qq.com) and Lincong Luo (18122747332@139.com).
